# ALDH2 gene polymorphism is associated with fitness in the elderly Japanese population

**DOI:** 10.1186/s40101-022-00312-1

**Published:** 2022-11-05

**Authors:** Kathleen Yasmin De Almeida, Mika Saito, Hiroki Homma, Yukina Mochizuki, Aoto Saito, Minoru Deguchi, Ayumu Kozuma, Takanobu Okamoto, Koichi Nakazato, Naoki Kikuchi

**Affiliations:** Graduate School of Health and Sport Science, Nippon Sport Science University, Tokyo, Japan

**Keywords:** Mitochondrial aldehyde dehydrogenase 2, Muscle strength, Sarcopenia, Mitochondrial ROS, Oxidative stress

## Abstract

**Purpose:**

The aldehyde dehydrogenase 2 (ALDH2) rs671 polymorphism, which is exclusive to the Asian population, is related to many diseases. A high reactive oxygen species production in mitochondria, and low muscle strength in athletes and non-athletes, has been observed, as our previous study demonstrated. The purpose of this research was to investigate the influence of *ALDH2* rs671 on the loss of muscle strength with aging and replicate our previous study in non-athletes.

**Methods:**

Healthy Japanese individuals (*n* = 1804) aged 23–94 years were genotyped using DNA extracted from saliva. Muscle strength was assessed using grip strength and chair stand test (CST). The interaction between age and genotypes was analyzed by two-way analysis of covariance (ANCOVA) adjusted for sex, body mass index (BMI), and exercise habit.

**Results:**

Individuals aged ≧55 with the AA genotype had a lower performance than those with the GG + GA genotype in the grip strength test (28.1 ± 9.1 kg vs. 29.1 ± 8.3 kg, *p* = 0.021). There was an interaction between age and genotype, where individuals with ≧55 years old AA genotype had a higher loss of strength compared to GG + GA genotypes in the CST (0.025). No interaction in other models and no sex differences were found.

**Conclusion:**

This study replicated previous results of the relationship between the AA genotype with lower muscle strength and as a novelty showed that this genotype is associated with a higher age-related loss of strength.

## Background

Muscle strength, an important factor for health, is necessary for daily activities and athletic performance. The loss of muscle mass and function (referred to as sarcopenia) with aging can be attributed to several factors, including oxidative stress [1]. The ability to deal with high levels of reactive oxygen species (ROS) [2] and reactive nitrogen species (RNS) is compromised with advancing age, impairing the cellular homeostasis of the muscle [2, 3].

During aging, a decrease in mitochondrial number and function is observed, which is pointed out as one of the main factors for the increase in ROS generation and cellular apoptosis, leading to the reduction of muscle fibers and causing oxidative damage in cells and, therefore, contributing for age-related muscle change [4–6]. Moreover, ROS accumulation causes change in the excitation–contraction coupling that engender a strength deficit in elderly individuals [1] and also impair the mitochondrial function provoking a sequence of reciprocal cause and effect [4, 6].

Several studies have suggested that loss of muscle strength is also associated with genetic factors. According to a meta-analysis, the heritability of muscle strength-related phenotypes (H2-msp) was estimated to be 52% [7]. The same study also showed that H2-msp can be related to sex and age; however, studies have reported that genetic differences can lead to a faster decline in the generation of muscle strength due to aging [8–10].

The aldehyde dehydrogenase 2 (*ALDH2)* rs671 polymorphism is considered exclusive to the Asian population. It is reported to be carried by up to 50% of individuals in East Asia [11]. *ALDH2* rs671 is a single-nucleotide polymorphism (SNP) in which a guanine is replaced by adenine leading to an amino acid change of glutamic acid to lysine. The substitution creates two possible alleles, G and A. Importantly, the amino acid change from glutamic acid to lysine impairs ALDH2 activity. A decrease in the activity of this enzyme impairs the metabolism of aldehyde, which is toxic to the organism, causing a high presence of acetaldehyde in the blood and alcohol flushing response [12, 13].

Previous studies have linked this polymorphism to hypertension, cancer, and body mass index [13–15]. Furthermore, a study by Wakabayashi et al. [16] showed a correlation between *ALDH2* rs671 and a deleterious effect in a mouse model, leading to a greater production of ROS in mitochondria. Our recent study analyzing the frequencies of *ALDH2* rs671 in different categories of Japanese athletes indicated that the mutation that causes the loss of enzymatic activity of ALDH2 is underrepresented in athletes compared to the general population and seems to be associated with lower muscle strength [17]; however, the aging effect was not analyzed.

Considering this, we hypothesized that loss of muscle strength with aging might depend on the *ALDH2* rs671 AA genotype. Therefore, in addition to replicating our previous *ALDH2* study in the general population [17], the novel aim of the present study is to assess the influence of *ALDH2* rs671 on the maintenance or loss of muscle strength with aging.

## Methods

### Subjects

The group of subjects studied comprised 1804 healthy Japanese individuals aged between 23 and 94 years. Among these, 1106 were women and 698 were men. The subjects in the present study were different from those in our previous study [17], and for the analysis, they were divided into two groups according to their age: < 55 years old and ≥ 55 years old. The subjects completed a questionnaire regarding their exercise habits and comprised healthy Japanese subjects from Tokyo and surrounding areas. DNA from saliva samples of these subjects was obtained between 2015 and 2019. The individuals participated voluntarily and provided written informed consent, which was approved by the ethics committee of Nippon Sport Science University. This study was conducted in accordance with the principles of the Declaration of Helsinki for research in humans.

Body weight and height were recorded, and body mass index (BMI) was calculated as weight (kg) divided by the square of the height (m). Systolic blood pressure and diastolic blood pressure were measured at a supine position using a vascular testing device (Omron Healthcare, Tokyo, Japan). The subjects completed original questionnaires on exercise habits, which included questions related to the type, duration, and frequency of activity. The options for responses regarding the frequency of activity were as follows: “never,” “once a week,” or “ ≥ twice a week.” The subjects who answered “ ≥ twice a week” were considered to have exercise habits.

### Genotyping

DNA was extracted from the saliva using an Oragene-DNA Kit (DNA Genotek, Ontario, Canada) according to the manufacturer’s instructions. The *ALDH2* rs671 polymorphism was identified using the TaqMan SNP Genotyping Assay (C__590093_1_) and the TaqMan Real-Time Polymerase Chain Reaction (PCR) system (Applied Biosystems, Foster City, CA, USA).

### Muscle strength assessment

Grip strength was assessed to evaluate upper extremity muscle strength using a hand grip dynamometer (Takei Scientific Instruments Co. Ltd., Tokyo, Japan). The subjects were allowed 3 attempts to attain the highest possible rating. The chair stand test was performed to evaluate lower-extremity muscle function. For this test, subjects were asked to sit on half of the chair surface with a straightened back and wrists crossed in front of the chest. The subjects were then asked to alternately stand and sit repeatedly for 30 s, and the number of times subjects could come to a full standing position was counted and recorded.

### Statistics

Analysis was performed using the SPSS statistical package version 25.0 for Mac (SPSS Inc., Chicago, IL, USA). Hardy–Weinberg equilibrium was assessed using the Pearson’s *χ*^2^ test on the observed genetic frequencies. The differences in age, height, weight, BMI, exercise habits, and smoking habits among the ALDH2 GG, GA, and AA genotypes were tested using ANOVA and Pearson’s *χ*^2^ test in both age groups. The interaction between the polymorphism and age group (< 55 age group vs. ≥ 55 age group) on grip strength and chair stand-up test was examined using a 2-way analysis of covariance (ANCOVA) adjusted for sex, BMI, and exercise habit. *P* values of < 0.05 were considered statistically significant.

## Results

The population was in Hardy–Weinberg equilibrium (*p* = 0.19) for this polymorphism, with genotypic frequencies of 7.5% for AA, 35.7% for GA, and 56.8% for GG, and a minor allele frequency of 25.4%.

Characteristics and fitness of the study subjects in the < 55 age group are shown in Table 1, categorized by *ALDH2* genotype. No differences were found in these characteristics between individuals with different genotypes.

**Table 1:** Association between ALDH2 genotype and grip strength and chair stand test in the < 55 age groups

	Genotype			Dominant	Recessive	*P* value		
	GG *n* = 544	GA *n* = 371	AA *n* = 75	AA + GA *N* = 446	GA + GG *N* = 915	Genotype	Dominant	Recessive
Women, n(%)	339 (62)	215 (58)	45 (60)	260 (58)	554 (61)	0.413*	0.112*	0.926*
Age, year	43.1 ± 8.2	43.0 ± 8.0	42.8 ± 8.2	42.9 ± 8.1	43.1 ± 8.1	0.932	0.714	0.852
Hight, cm	163.9 ± 8.2	164.3 ± 8.1	163.5 ± 8.1	164.2 ± 8.2	164.1 ± 8.2	0.676	0.618	0.589
Weight, kg	60.5 ± 11.9	61.8 ± 12.9	61.5 ± 9.9	61.8 ± 12.4	61.0 ± 12.4	0.269	0.108	0.681
BMI, kg•m^−2^	22.4 ± 3.3	22.7 ± 3.4	23.0 ± 3.1	22.8 ± 3.4	22.5 ± 3.3	0.152	0.064	0.245
Systolic blood pressure, mmHg	120.8 ± 16.7	120.9 ± 14.8	121.0 ± 15.9	120.9 ± 15.0	120.8 ± 15.9	0.992	0.906	0.935
Diastolic blood pressure, mmHg	71.2 ± 11.6	70.6 ± 11.3	71.7 ± 10.9	70.8 ± 11.2	71.0 ± 11.5	0.672	0.586	0.602
Exercise habit, yes (%)	291(53)	179(48)	39(52)	218(49)	470(51)	0.295*	0.469*	0.916*
Grip Strength, kg	33.7 ± 9.0	34.6 ± 10.0	33.8 ± 8.9	34.5 ± 9.8	34.1 ± 9.5	0.566†	0.895†	0.335†
Chair stand test, times	28.0 ± 5.8	27.6 ± 5.4	28.4 ± 5.5	27.8 ± 5.4	27.9 ± 5.6	0.473†	0.612†	0.370†

Mean ± SD. *BMI* body mass index.

*Pearson’s *χ*^2^ test

^†^ANCOVA adjusted by age, sex, and exercise habit

For subjects in the ≥ 55 years old group, there was a significant difference in the BMI, individuals with AA genotype had a higher index (kg•m^−2^) compared to GG and GA genotypes (GG: 22.4 ± 2.9; GA: 22.0 ± 2.7; AA: 23.0 ± 2.9, *p* = 0.035) (Table 2). There was a significant difference in the grip strength test, where individuals with the ALDH2 AA genotype performed the worst results in the recessive model (28.1 ± 9.1 kg for AA genotype, and 29.1 ± 8.3 kg for GG + GA genotypes, *p* = 0.021) (Table 2).

**Table 2:** Association between ALDH2 genotype and grip strength and chair stand test in the ≥ 55 age groups

	Genotype			Dominant	Recessive	*P* value		
	GG *n* = 481	GA *n* = 272	AA *n* = 61	AA + GA *n* = 333	GA + GG *N* = 753	Genotype	Dominant	Recessive
Women, *n* (%)	311 (65)	160 (59)	36 (59)	196 (59)	471 (63)	0.245*	0.198*	0.704*
Age, year	68.8 ± 7.9	69.4 ± 8.3	70.6 ± 8.5	69.6 ± 8.3	69.1 ± 8.1	0.217	0.143	0.091
Hight, cm	158.4 ± 8.9	159.5 ± 8.4	158.2 ± 8.4	159.2 ± 8.4	158.8 ± 8.6	0.233	0.183	0.529
Weight, kg	56.6 ± 10.4	56.2 ± 9.3	57.8 ± 9.2	56.4 ± 9.3	56.5 ± 9.9	0.543	0.865	0.356
BMI, kg•m^−2^	22.4 ± 2.9	22.0 ± 2.7	23.0 ± 2.9	22.2 ± 2.8	22.3 ± 2.8	0.035	0.277	0.075
Systolic blood pressure, mmHg	135.6 ± 18.6	134.6 ± 18.2	137.7 ± 20.7	135.3 ± 18.7	135.3 ± 18.5	0.491	0.854	0.280
Diastolic blood pressure	78.0 ± 11.8	77.5 ± 12.8	78.4 ± 11.2	77.7 ± 12.5	77.9 ± 11.9	0.810	0.812	0.495
Exercise habit, yes (%)	372 (77)	216 (79)	49 (80)	265 (80)	588 (78)	0.739*	0.148*	0.734*
Grip strength, kg	28.9 ± 8.5	29.3 ± 7.9	28.1 ± 9.1	29.0 ± 8.0	29.1 ± 8.3	0.138†	0.245†	0.021†
Chair stand test, times	24.8 ± 6.3	24.3 ± 6.3	23.2 ± 7.2	24.0 ± 6.4	24.6 ± 6.3	0.270†	0.089†	0.124†

Mean ± SD. *BMI* body mass index.

*Pearson’s *χ*^2^ test

^†^ANCOVA adjusted by age, sex, and exercise habit

**Fig. 1:**
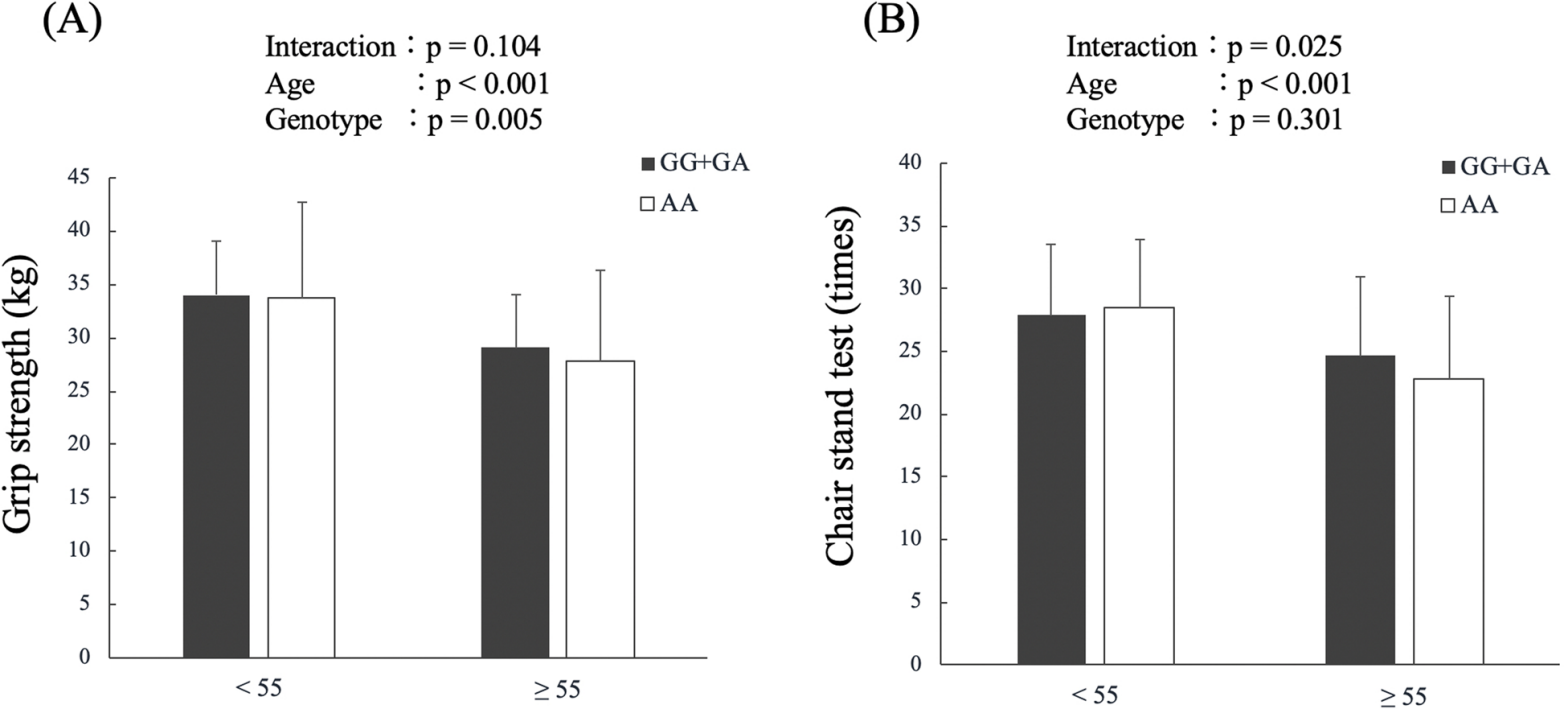
Relationship between the aldehyde dehydrogenase 2 (ALDH2) genotypes and age group for the grip strength (**A**) and chair stand test (**B**)

There was a significant interaction between the ALDH2 genotypes and age in the chair stand test, where individuals aged ≥ 55 years with the AA genotype had a higher loss of strength when compared with individuals with the GG + GA genotypes (*p* = 0.025) as shown in Fig. 1B. However, the same interaction was not seen for the grip strength test (*p* > 0.05, Fig. 1A). There was no significant interaction for the other models in the CST (genotypes AA vs. GA vs. GG, interaction *p* = 0.145, genotype *p* = 0.199, age *p* < 0.001; dominant model AA + GA vs. GG, interaction *p* = 0.212, genotype *p* = 0.069, age *p* =  < 0.001) and grip strength (genotype AA vs. GA vs. GG interaction *p* = 0.260, genotype *p* = 0.044, age *p* < 0.001; dominant model AA + GA vs. GG interaction *p* = 0.162, genotype *p* = 0.268, age *p* < 0.001).

In Table 3, it is possible to see the subjects’ characteristics according to sex, where only in women it was seen a significant difference in weight and BMI considering ALDH2 genotypes (*p* = 0.016 and *p* = 0.002, respectively). Differences between sex were also analyzed regarding the performance. Men presented a better performance in all genotypes, in both grip strength and CST, but the differences were not statistically relevant in none of the analyzed models (Table 3).

**Table 3:** Association between ALDH2 genotypes in grip strength and chair stand test dividing by sex

		Genotype			Dominant	Recessive	*P* value		
		AA	GA	GG	AA + GA	GA + GG	Genotype	Dominant	Recessive
Men	*n*	55	268	375	323	644			
	Age, year	53.7 ± 16.1	53.3 ± 16.9	53.4 ± 15.5	53.4 ± 16.1	53.4 ± 16.7	0.985	0.974	0.872
	Hight, cm	168.8 ± 6.1	169.6 ± 6	170 ± 6.5	169.8 ± 6.3	169.4 ± 6	0.373	0.255	0.212
	Weight, kg	65.8 ± 8	68 ± 11	68.3 ± 10.1	68.1 ± 10.5	67.6 ± 10.6	0.24	0.391	0.097
	BMI, kg•m-2	23.1 ± 2.5	23.6 ± 3.1	23.6 ± 2.9	23.6 ± 3	23.5 ± 3	0.464	0.724	0.237
	Grip strength, kg	39.9 ± 7.2	41.3 ± 7.3	41.1 ± 6.7	41 ± 7.3	41.2 ± 7	0.381	0.105	0.921
	Chair stand test, times	27 ± 7.2	26.8 ± 6	27.3 ± 5.9	27.3 ± 5.9	27.1 ± 6	0.562	0.506	0.291
Women	n	81	375	650	456	1025			
	Age, year	56.4 ± 16.2	54.7 ± 14.2	56.2 ± 14.8	55 ± 14.6	55.7 ± 14.6	0.25	0.17	0.667
	Hight, cm	156 ± 5.8	157 ± 5.9	156.3 ± 5.8	156.8 ± 5.9	156.6 ± 5.8	0.123	0.125	0.445
	Weight, kg	55.8 ± 8.7	53.3 ± 8	53.1 ± 7.9	53.7 ± 8.2	53.1 ± 7.9	0.016	0.004	0.172
	BMI, kg•m-2	22.9 ± 3.3	21.6 ± 3	21.7 ± 3	21.8 ± 3.1	21.7 ± 3	0.002	< 0.001	0.515
	Grip strength, kg	25.3 ± 5.1	26 ± 4.4	25.9 ± 4.5	25.8 ± 4.6	25.9 ± 4.4	0.595	0.503	0.343
	Chair stand test, times	25.5 ± 6.5	25.8 ± 6	26.1 ± 6.4	25.7 ± 6.1	26 ± 6.3	0.51	0.246	0.651

Mean ± SD. *BMI* body mass index

## Discussion

The data indicated that a greater age-related (≥ 55) loss of muscle strength occurs in individuals carrying the AA genotype, showing a relationship between the genotypes of the *ALDH2* polymorphism and strength, where the A allele is related to less muscle strength, especially in older populations. In addition, our data were replicated in a previous study. Therefore, the *ALDH2* rs671 polymorphism is associated with muscle phenotypes in Japanese subjects, particularly in older populations.

Muscular strength loss due to age can occur at a certain rate that is approximated at 12 to 14% per decade, according to Hurley and Ruth [18]. In this same study, it was also pointed out that there’s a functional decline of strength at the age of about 50 years old [18]. One of our previous studies also identified this same characteristic of strength loss due to age, especially for ≥ 55 years old, and associated with some polymorphisms, such as *ACTN3* R577X [9]. Therefore, this was an interesting way to divide groups of the study’s subjects and is indicated for further analysis.

The first study to verify the association between polymorphisms and strength in an Asian population included a total of 3055 subjects, 1714 athletes, and 1341 healthy control subjects (non-athletes). The study pointed out that individuals with different *ALDH2* rs671 polymorphism genotypes presented different results in muscle strength tests, where the AA genotype and the A allele were associated with poorer results in both grip strength and chair stand tests. In addition, the AA genotype also occurs at a significantly lower frequency in athletes than in non-athletes, indicating an association with sports performance [17].

ALDH2 deficiency is associated with an increase in ROS production through mitochondrial respiration [16] and reduced muscle mass in mice [19] and is suggested to impair mitochondrial function and promote muscle loss [16, 17, 19]. A study by Kobayashi et al. [20] in a mouse model, showed that the mutation in *ALDH2* leads to muscle atrophy due to the accumulation of muscle oxidative stress, decreased expression of anabolic and catabolic muscle factors, and decreased body weight in comparison to the wild type [20]. Histological analysis showed a significantly smaller muscle fiber diameter compared to that of wild-type mice, suggesting that the reduced muscle weight observed in mice with ALDH2 deficiency was a consequence of muscle atrophy [20].

In mice, the polymorphism leads to osteoblastic dysfunction due to oxidative stress by the accumulation of acetaldehyde, even in the absence of alcohol ingestion [21], similar to the effects found by Hoshi and collaborators [22], where ALDH2 transgenic mice had severe osteoporotic phenotypes and high aldehyde levels with no alcohol intake [22]. The polymorphism was also identified as a contributor to Alzheimer’s disease progression, as risk factors, such as mitochondrial dysfunction, oxidative stress, and increased aldehyde levels in the brain, are found at greater levels in *ALDH2*-mutated mice compared with those in wild-type mice [2].

A recent study by Kasai and collaborators [23] showed that in aged mice with an ALDH2 deficiency, the cross-sectional area of fast muscle fibers of the soleus is reduced. Fast muscle fibers are rich in mitochondria, and the cross-sectional area of type II fibers is reduced with aging, while type I fibers are less affected [24]. Hence, it is suggested that ALDH2 deficiency accelerates age-related muscle loss, especially in the muscles rich in mitochondria, accelerating muscle atrophy due to aging and oxidative stress in fast muscle fibers.

Therefore, in addition to the loss of ability to deal with ROS and inherent changes in the composition and architecture of muscle and contractile filaments due to aging [25] which explains why the results are already different between individuals < 55 and ≧55 years, the *ALDH2* polymorphism also seems to increase mitochondrial ROS production [16]. This leads to greater oxidative stress and subsequently to muscle atrophy [20], resulting in an amplified additive effect of strength loss, as demonstrated by the results.

The genetic inactivation of the aldehyde dehydrogenase 2 in the mutant allele (A) is reported to be dominant over the wild-type allele G. Studies show that the heterozygous for this polymorphism (GA genotype) presents a 40 to 60% reduction of ALDH2 enzymatic activity [26–28], and in an investigation in Japanese subjects, it was seen that the effects of the polymorphism on alcohol-associated manifestations was obviously more marked in the homozygous for the mutation (AA individuals) compared to heterozygous, and so was the drinking frequency and amounts of alcohol consumption that decreased following the order GG – GA – AA, and are pointed as two indicators of alcohol sensitivity that can be a consequence of the effects of the enzyme deficiency [29]. Therefore, the partial and not complete loss of the enzymatic activity in heterozygotes may be the reason why our results showed a higher effect of strength loss associated with only the AA homozygotes.

Although health maintenance with aging is multifactorial, a very important contributing factor is the preservation of physical health, especially that of the muscles and bones. The loss of muscle function impairs many daily activities and renders the elderly more dependent on others, in addition to being a serious risk factor for falls, fractures, and many diseases that can be caused or aggravated by sarcopenia. Thus, studies that help to identify the factors that affect the health of this population are essential for gaining a better understanding of the relevant risks and predispositions in order to create strategies to preserve the quality of life of these individuals.

However, our study had some limitations. First, we could not control for external factors such as the presence of comorbidities or medications, any of which could have influenced the results of our battery of tests. In addition, our measurements for the battery of tests might have been more sensitive for middle-aged and elderly subjects (age ≥ 55 years) than for younger subjects (age < 55 years). Moreover, our results showed that, in women, the ALDH2 genotypes might influence a higher weight and BMI. However, we understand these results as an effect of the low sample size, and future studies are necessary to analyze the effect of the polymorphism in weight and BMI considering sex differences, and its influence in muscle strength. Besides that, other polymorphisms related to muscle strength loss were not investigated. An association study with a larger gene panel, evaluating not only genotypic and allelic frequencies but also other aspects, such as gene expression and association between polymorphisms, would contribute to a better understanding of the genetic factors contributing to muscle strength loss in a broader and more representative way. Finally, this study lacked additional information about the studied individuals, such as sequelae from previous pathologies and other factors that could, in some way, affect the results, especially in individuals aged 55 years or older.

## Conclusions

Our study suggests that the loss of muscle strength due to age can be aggravated by the genotype of the *ALDH2* rs671 polymorphism, where the presence of the AA genotype is detrimental to the maintenance of strength in older individuals.

## Data Availability

The study’s data is available from the corresponding author upon reasonable request.

## Abbreviations

ALDH2: Aldehyde dehydrogenase 2
ANCOVA: Analysis of covariance
BMI: Body mass index
CST: Chair stand test
DNA: Deoxyribonucleic acid
H2-msp: Heritability of muscle strength-related phenotypes
PCR: Polymerase chain reaction
RNS: Reactive nitrogen species
ROS: Reactive oxygen species
SD: Standard deviation
SNP: Single-nucleotide polymorphism
